# Supplementary material for: Mapping and targeting of C1ql1-expressing cells in the mouse
Source: Sci Rep. 2023 Oct 16;13:17563. doi: 10.1038/s41598-023-42924-2 (PMC10579299; doi:10.1038/s41598-023-42924-2)
Supplement: Supplementary file 1 — Supplementary Figures. [file 41598_2023_42924_MOESM1_ESM.pdf]

Title: Mapping and targeting of *C1ql1*-expressing cells in the mouse

Authors:

Shayan Moghimyfiroozabad[1], Maëla A. Paul[1], Séverine M. Sigoillot[1,2]✉, Fekrije Selimi[1,2]✉

Affiliations:

[1]Center for Interdisciplinary Research in Biology (CIRB), College de France, CNRS, INSERM, Université PSL, Paris, France

[2]These authors contributed equally: Séverine M. Sigoillot and Fekrije Selimi

✉Corresponding authors: Fekrije Selimi and Séverine M. Sigoillot

This PDF file includes:

Supplemental Figure 1, 2 and 3

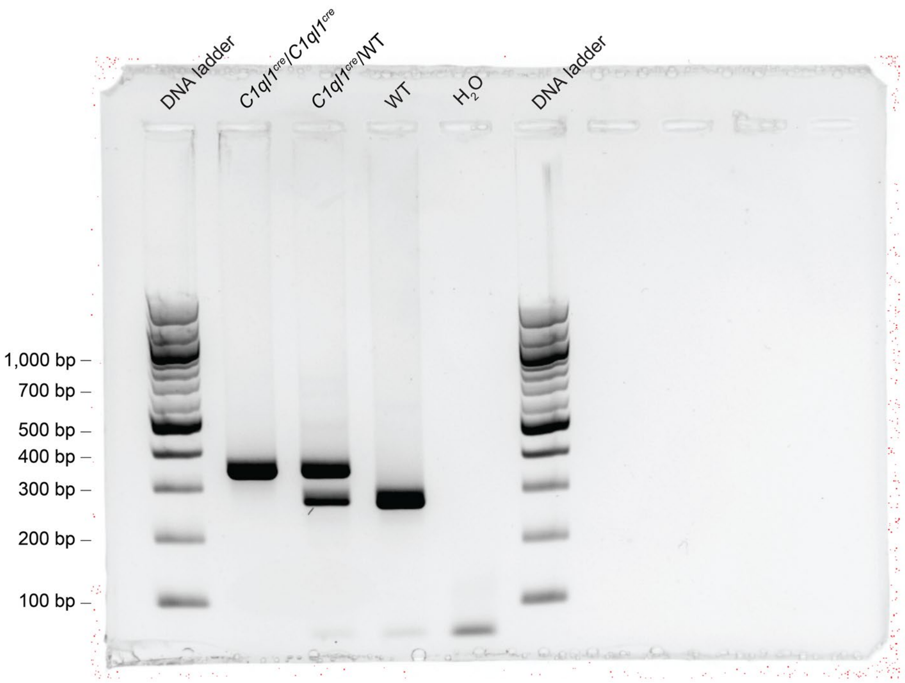

Supp Fig 1. Original gel of the representative cropped image presented in Fig 1B.

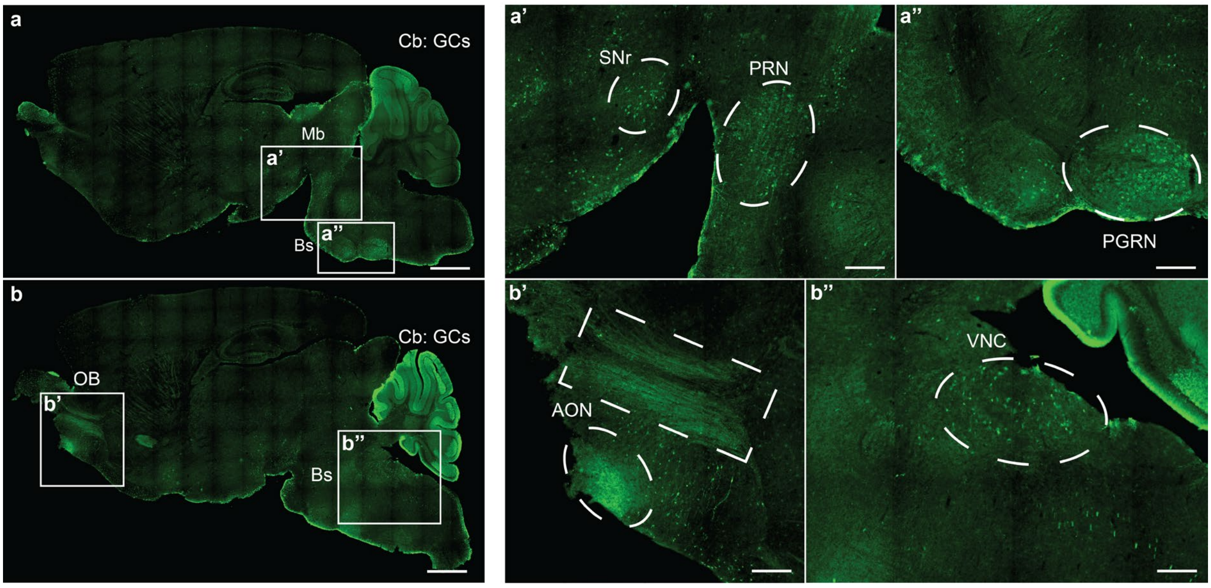

Supp Fig 2. Reproducibility of the targeting capacity of the *C1ql1^cre^* mouse line.

Medial to lateral sagittal sections from postnatal day 28 (P28) $C1ql1^{cre}/R26^{R\text{-}EYFP}$ mouse brain were immunostained for EYFP using an anti-GFP antibody. Mosaic images were acquired using a Zeiss Axiozoom V16 macroscope (a – b). EYFP was highly expressed in the cerebellum (Cb), in particular in granule cells (GCs) and in the brainstem (Bs), and present in other nuclei from the midbrain (Mb) and olfactory bulb (OB). Scale bars = 1000 µm. (a' – b'') High magnification of different nuclei or neurons expressing EYFP in P28 $C1ql1^{cre}/R26^{R\text{-}EYFP}$ mice. Scale bars = 250 µm. AON: Anterior Olfactory Nucleus, PGRN: Paragigantocellular Reticular Nucleus, PRN: Pontine Reticular Nucleus, SNr: Substantia Nigra pars reticulata, VNC: Vestibular Nuclei.

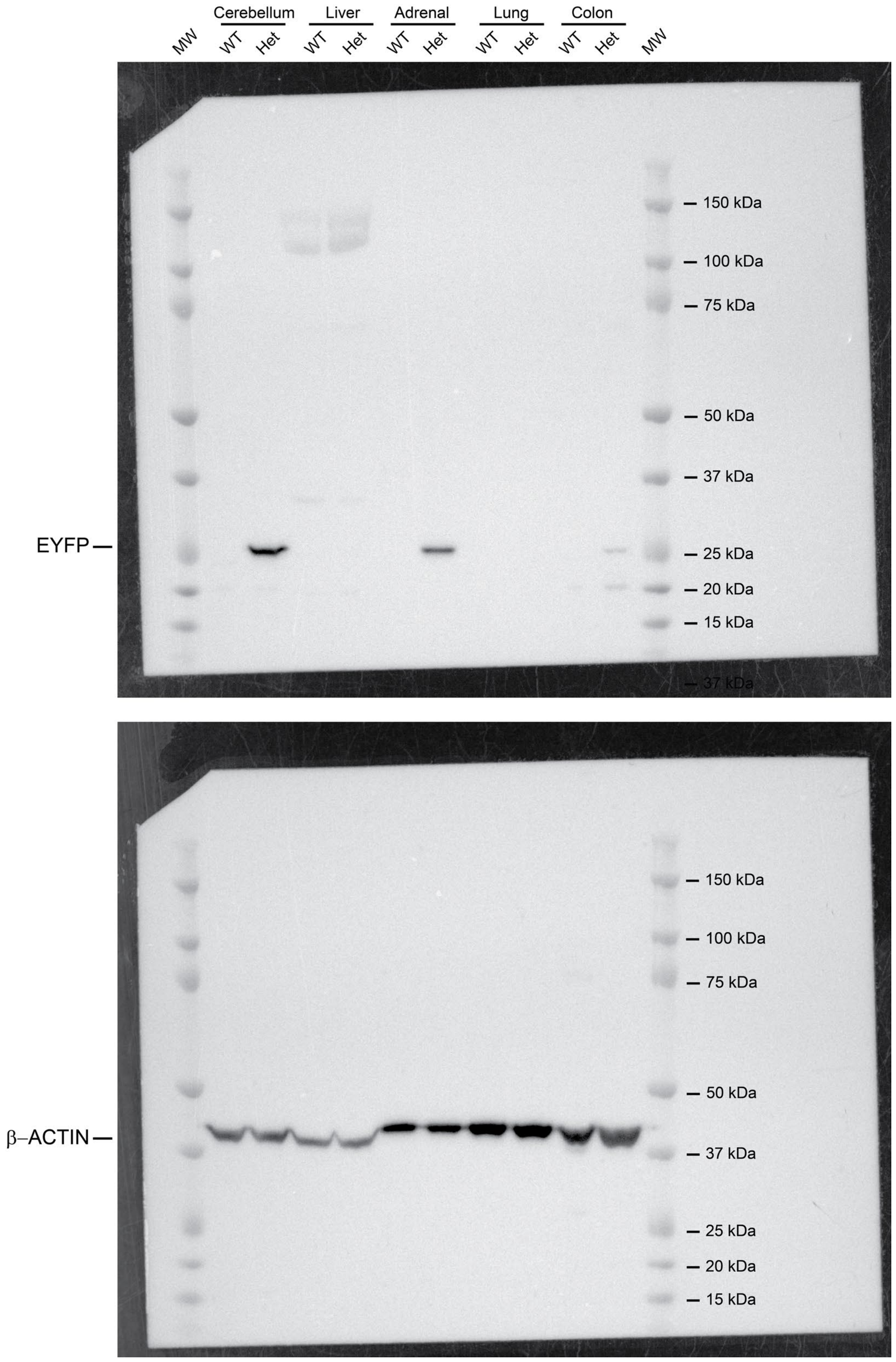

Supp Fig 3. Original western blots of the representative cropped image presented in Fig 2A.

Het: Heterozygote, MW: Molecular weight, WT: Wild-type.
